# A social ecology of rectal microbicide acceptability among young men who have sex with men and transgender women in Thailand

**DOI:** 10.7448/IAS.16.1.18476

**Published:** 2013-08-01

**Authors:** Peter A Newman, Surachet Roungprakhon, Suchon Tepjan

**Affiliations:** 1 Factor-Inwentash Faculty of Social Work, University of Toronto, Ontario, Canada; 2 Department of Computer Technology, Faculty of Science and Technology, Rajamangala University of Technology Phra Kakhon, Bangkok, Thailand

**Keywords:** HIV prevention, rectal microbicide, acceptability of healthcare, social ecological model, gay men, transgender women, Thailand, qualitative research

## Abstract

**Introduction:**

With HIV-incidence among men who have sex with men (MSM) in Bangkok among the highest in the world, a topical rectal microbicide would be a tremendous asset to prevention. Nevertheless, ubiquitous gaps between clinical trial efficacy and real-world effectiveness of existing HIV preventive interventions highlight the need to address multi-level factors that may impact on rectal microbicide implementation. We explored the social ecology of rectal microbicide acceptability among MSM and transgender women in Chiang Mai and Pattaya, Thailand.

**Methods:**

We used a qualitative approach guided by a social ecological model. Five focus groups were conducted in Thai using a semi-structured interview guide. All interviews were digitally recorded, transcribed verbatim in Thai and translated into English. We conducted thematic analysis using line-by-line and axial coding and a constant comparative method. Transcripts and codes were uploaded into a customized database programmed in Microsoft Access. We then used content analysis to calculate theme frequencies by group, and Chi-square tests and Fisher's exact test to compare themes by sexual orientation/gender expression and age.

**Results:**

Participant's (*n*=37) mean age was 24.8 years (SD=4.2). The majority (70.3%) self-identified as gay, 24.3% transgender women. Product-level themes (side effects, formulation, efficacy, scent, etc.) accounted for 42%, individual (increased sexual risk, packaging/portability, timing/duration of protection) 29%, interpersonal (trust/communication, power/negotiation, stealth) 8% and social–structural (cost, access, community influence, stigma) 21% of total codes, with significant differences by sexual orientation/gender identity. The intersections of multi-level influences included product formulation and timing of use preferences contingent on interpersonal communication and partner type, in the context of constraints posed by stigma, venues for access and cost.

**Discussion:**

The intersecting influence of multi-level factors on rectal microbicide acceptability suggests that social–structural interventions to ensure widespread access, low cost and to mitigate stigma and discrimination against gay and other MSM and transgender women in the Thai health care system and broader society will support the effectiveness of rectal microbicides, in combination with other prevention technologies, in reducing HIV transmission. Education, outreach and small-group interventions that acknowledge differences between MSM and transgender women may support rectal microbicide implementation among most-at-risk populations in Thailand.

## Introduction

HIV-incidence among men who have sex with men (MSM) in Bangkok is among the highest in the world [1]. MSM in Thailand are at the forefront of a burgeoning and largely unchecked epidemic among MSM in Asia [2, 3]. As unprotected receptive anal intercourse has the highest per act risk of HIV acquisition among sexual behaviours [4], the introduction of an efficacious rectal microbicide (RM) would be a tremendous asset to global HIV prevention, particularly among MSM.

Topical microbicides are products designed to be applied to the vaginal or rectal mucosa to prevent or significantly reduce the risk of HIV acquisition. Recent clinical trials provide proof of concept that topical [5] or systemic use [6] of antiretroviral medications can significantly reduce HIV acquisition risk during unprotected vaginal or anal sex [7]. Yet generally poor adherence and compromised product effectiveness in the iPrEx oral pre-exposure prophylaxis (PrEP) trial [6] and the CAPRISA 004 vaginal microbicide trial [5] highlight the decisive role of social and behavioural factors.

Adherence is generally greater in the optimized realm of clinical trials – with assured product access, detailed product instructions, consistent counselling and testing, and financial incentives – in contrast to real-world use. Nevertheless, clinical trials represent an opportunity to better understand product effectiveness by integrating social and behavioural research throughout RM testing and development [7–9]. It is also crucial to conduct social–behavioural research outside the specialized domain of trials [10], including pre-clinical acceptability research conducted before Phase I trials are launched.

With RM development and testing among MSM in the early stages, we are at a strategic juncture for advancing the implementation science of RMs – in order to bridge expectable gaps between clinical trial efficacy and real-world effectiveness [11, 12]. Evidence of the influence of product- and user-related factors [13–17] and limited investigation of partner influence [8] on hypothetical RM acceptability, all among US MSM, suggest the importance of conducting RM acceptability research in low- and middle-income countries (LMIC) with the greatest burden of HIV – and assessing factors beyond the individual level. To that end, we explored the social ecology of RM acceptability among gay and bisexual men, and transgender women (TG) in Thailand.

## A social ecological approach

Social ecological models (SEM), based on Bronfenbrenner's [18] ecological systems theory, have been used successfully to broaden health research and interventions beyond the individual level [19, 20]. SEM provide an overarching conceptual framework for exploring multiple and intersecting levels of influence on human behaviour, incorporating individual, interpersonal and social–structural levels, with particular attention to social, institutional and cultural contexts [19].

A social ecological approach may be particularly warranted in the case of RMs – a product designed for rectal use to prevent a highly stigmatized infectious disease that disproportionately affects marginalized populations – to support effectiveness in preventing HIV infection (i.e. T2 implementation science) [12]. Low education, sex work, HIV and sexual stigma, and discrimination in health care documented among MSM and TG at elevated risk for HIV in Thailand [21, 22] suggest that a narrow focus on product- and individual-level factors absent attention to interpersonal and social–structural contexts risks significant gaps in the translation of future RMs into routine practice.

## Methods

### Setting

The study was conducted in July and August 2011 in collaboration with the main community based-organizations (CBOs) serving MSM and TG in Chiang Mai and Pattaya, Thailand. Mplus (Chiang Mai) and Service Workers in Group (SWING) (Pattaya) provide social support, recreational activities, HIV prevention education and outreach, the latter with a focus on male sex workers.

### Study design

We conducted a qualitative study given our objective of exploring the social ecology of RM acceptability in a novel population and setting. Thailand has been the site of several biomedical HIV prevention trials, including PrEP [6] and HIV vaccines (RV144) [23], with limited investigation of social–structural contexts in which these technologies would be introduced [24]. RM acceptability is also a relatively new research area with limited exploration of extra-individual factors that may constrain or enable product uptake.

The qualitative design enabled in-depth exploration of potential interpersonal and social–structural factors that may impact on future RM acceptability. We used focus groups, as opposed to individual interviews, to facilitate exploration of interpersonal and social dynamics beyond individual-level perspectives.

### Study participants

We conducted recruitment in each city through our CBO partners. Eligibility criteria were: being a self-identified gay or bisexual man, or TG woman, aged 18–30 years and fluent in Thai.

We included Thai MSM who self-identified as “gay,” “bisexual,” “gay *rook* ” (insertive partner), “gay *rub*” (receptive partner), “both” (*boat*) (versatile), “*dai mod* ” (literally, okay with anything) and others. “Transgender” in the Thai context includes biological males across a broad spectrum of female gender expression, dressing as women, female gender identification, taking hormones and planning or having engaged in gender reassignment surgery [25–27].

### Data collection

We conducted five focus groups using a semi-structured interview guide in Thai with scripted probes. Focus groups were segmented by age (18–24 years; 25–30 years) and sexual/gender identity (gay/bisexual; transgender) to encourage comfort and candour. Most group participants were familiar with one another as members of the CBOs, which focused on sexual health; this facilitated comfort in discussion of sex and HIV in a group setting.

The semi-structured interview guide was informed by SEM [18]. After eliciting awareness and knowledge regarding RMs, then providing a brief definition to ensure participants were addressing the intended product, questions explored product preferences and individual, interpersonal and social–structural influences on RM acceptability. The guide was created in English, translated into Thai, back-translated into English and revised in Thai. Questions were based on published RM acceptability research among MSM in the United States [8, 13–17] and on our previous investigations of social–structural contexts of HIV prevention among MSM and TG populations in Thailand [22, 24].

Sample questions and probes included: What features of the product would make you more or less likely to use it? Probes: formulation (gel, cream, suppository, other), colour, etc. In what ways might your relationship or the partner you are having sex with influence your use of RMs? Probes: impact on sexual pleasure, ability to conceal use, power dynamics, partner type, etc. Are there possible social or community concerns that might influence you or your friends’ use of an RM? Probes: stigma, access, prescription. Each focus group was 60–90 min in duration. All participants completed an anonymous, two-page socio-demographic questionnaire.

### Data analysis

All interviews were digitally recorded, transcribed verbatim in Thai and translated into English. We conducted thematic analysis in an iterative process and revised the interview guide to reflect emerging data and questions [28, 29]. Two team members independently analysed three transcripts using line-by-line coding, with the interview guide as an initial template, and open coding to identify new themes. We then met as a team to construct a codebook, which we applied in coding subsequent transcripts. Differences in coding were resolved by consensus.

We entered all transcripts and codes into a customized database programmed in Microsoft Access and organized data by codes. We then used axial coding and a constant comparative method to relate themes and categories to one another [29]. Axial coding is an intermediate stage in data analysis that focuses on the conditions, contexts, interactions and social processes that relate codes to each other [30]. Guided by SEM, we created a conceptual map depicting all categories and themes. We achieved theoretical saturation in that no new themes emerged in the final two focus groups.

We applied content analysis by calculating theme frequencies by group. We used Chi-square tests and Fisher's exact test where contingency table cell counts were less than 5, to compare themes by age and MSM/TG populations. Data source triangulation (across focus groups) and methodological triangulation (thematic and content analysis) enhance the validity of the findings [31, 32].

### Ethical considerations

The study was approved by the University of Toronto Research Ethics Board, with administrative approvals from CBO partners. All participants provided written informed consent. Research staff received research ethics training and signed confidentiality agreements.

## Results

Participant's (*n*=37) mean age was 24.8 years (SD=4.2). The majority (70.3%) self-identified as gay, 24.3% TG. Table 1 describes participant socio-demographics.

**Table 1:** Socio-demographic characteristics of study participants, Chiang Mai and Pattaya, Thailand (*n*=37)

Characteristic	*n*	%
Age (years) mean (SD)	24.8 (4.2)	
Sexual orientation		
Gay	26	70.3
Bisexual	2	5.4
Transgender women	9	24.3
Education		
6th grade or less	3	8.1
9th grade	4	10.8
High-school degree	11	29.7
Bachelor's degree or higher	19	51.3
Employment		
Full-time	14	37.8
Part-time	13	35.1
Unemployed	10	27.0
Income (monthly, THB)		
≤5,000	9	24.3
5,001–10,000	12	32.4
10,001–15,000	12	32.4
15,001–20,000	4	10.8

SD=standard deviation; THB=Thai Baht (THB 5,000=USD 163).

Based on content analysis, product themes accounted for 42%, individual 29%, interpersonal 8% and social–structural 21% of total codes. Table 2 indicates theme (in rank order) and category frequencies by focus group and across groups. Overall, we identified significant differences in content by sexual orientation/gender identity (χ^2^=10.56, *p*=0.014); individual and social–structural themes occurred more frequently among MSM, product and interpersonal themes more frequently among TG participants. Sub-theme frequencies differed by MSM/TG within individual (*p*=0.001), interpersonal (*p*=0.027) and social–structural (*p*=0.018) levels. Theme frequencies did not differ by age group.

**Table 2:** Rectal microbicide acceptability categories and themes by focus group (*n*=37)

Focus group (*n*)	1 (*n*=8)	2 (*n*=9)	3 (*n*=8)	4 (*n*=6)	5 (*n*=6)	
Population	MSM	MSM	TG	MSM	MSM	
Age range (years)	18–24	25–30	18–30	25–30	18–24	
City	Chiang Mai	Chiang Mai	Chiang Mai	Pattaya	Pattaya	

Categories and themes	Frequency					Total
Product						
Side effects	5	38	23	24	24	114
Formulation	8	23	20	15	12	78
Efficacy/quality	2	10	21	16	17	66
Scent	6	9	16	7	5	43
Applicator	10	6	7	11	3	37
Colour	7	7	9	4	5	32
Taste	3	6	10	7	4	30
STI protection	1	5	2	4	4	16
Subtotal	42	104	108	88	74	416
Individual						
Sexual risk behavior	13	16	30	28	14	101
Packaging/portability	8	19	13	17	19	76
Timing/duration	6	22	8	20	18	74
Feel/leakage	5	4	1	5	8	23
Volume	2			6	7	15
Subtotal	34	61	52	76	66	289
Interpersonal						
Trust and communication	7	11	7	10	6	41
Power/negotiation	2	3	13	4	7	29
Stealth	4	2	2	1	3	12
Subtotal	13	16	22	15	16	82
Social and structural						
Cost	8	15	8	18	12	61
Access	7	15	12	14	12	60
Community influence	5	2	11	15	1	34
Stigma	4	1	4	12	7	28
Media/promotion	11	6		1	4	22
Subtotal	35	39	35	60	36	205
Total	124	220	217	239	192	992

MSM=men who have sex with men; TG=transgender women; STI=sexually transmitted infections.

### Product

#### Side effects: “Could it cause … cancer?”

Side effects was the most prevalent theme overall, reflecting concerns about unknown effects of RM use over time. Participants were largely accepting of minor side effects as a trade-off for protection against HIV infection – “If we use it for a long time and our anus get darker, that I can accept it because it is inside, right?” (Focus Group [FG] 4) – but concerned about side effects that might accrue over time: “If it can prevent 100% and only anal area gets dark, it's amazing; but if after five years rashes and itchiness happen, I wouldn't accept it” (FG2).

Of greatest concern were fears of cancer, noted in every group: “Could it possibly cause serious side effects like cancer?” (FG1); “I'm afraid of anal cancer” (FG3). A trade-off between HIV and cancer was described as unacceptable: “I would rather be HIV infected than have cancer. There is medication for HIV; but cancer … counting down the days to death” (FG5).

Some participants indicated concern about developing resistance to ART – “If time passes, it might cause drug resistance” (FG1) – fearing that they might contract HIV – “It can also make us drug resistant to the medication if we get infected” (FG3).

#### Formulation: “Make it in gel form …”

Participants generally indicated preference for a gel, based on familiarity and convenience, rather than other formulations: “make it in gel form because gay people have already got gel for lubricant” (FG1). “In my opinion I think it would be nice if it is made in gel form because normally we have sex every day and we need to use a gel-based lubricant” (FG4).

Some participants indicated alternative and conflicting preferences: “like a suppository, I feel like it can go deeper and I feel safer” (FG3); but, “if it's in suppository form nobody will insert it before having sex” (FG4). Another participant noted, “I would rather use a thick cream, not gel. Gel tends to leak … undesired watery leak” (FG3).

#### Efficacy: “It must work 100%”

The need for assurances about efficacy and product quality was evidenced in every group. A participant explained, “Because preventing HIV is important, it needs to be successful first” (FG4); however, he also revealed the widespread expectation of 100% efficacy: “If we use it to prevent HIV disease, it must work 100%” (FG4). “At first no one would trust this RM as 100%; it would take time” (FG4), noted another participant.

Participant interactions indicated mixed reactions to the facilitator's raising the possibility of a partial efficacy RM. Some aspired to joint condom and RM use, others to replacing condoms with an RM: “I will still use it together with a condom; because 50% doesn't really help much” (FG3); “For those who don't usually like to use condoms, having this at 50% effective would be like a treat for them!” (FG3). Other described maintaining condom use with no RM uptake: “I won't use it; it's like a burden. Using condoms is already one thing …” (FG4); “This increases the cost of having sex, too” (FG4).

An additional concern was the extent to which an RM would be protective for insertive as well as receptive partners in anal sex: “So it would be good for those who are sexually receptive? What about those who are active? Would tops be protected from bottoms?” (FG1).

#### Scent: “Please, no banana!”

Scent preferences ranged from no scent, to aversion to medicinal scents, to preferences (and aversions) for scents of various fruits, flowers, perfumes and herbs. In addition to a preference for no scent – “Personally, I don't like smell” (FG1); “I think no one would want to use it” (FG2) – others indicated, “If it had a smell like a hospital, it's not good to use it” (FG4). Scent preferences included, “fruity like strawberry, banana or orange” (FG5); “I'd be likely to use it if it's the light smell of a flower; a nice smell, not like banana …!”; and “Oh no … please, no banana!” (FG3).

#### Colour: “If it leaks, it won't look so pretty”

Colour preferences included aesthetic as well as pragmatic concerns – being able to ensure one had applied the product, unpleasant looking leakage and differentiation from existing lubricants – as well as an understanding that individual preferences may differ. “I think light colour would be good; if it has no colour, we can't know whether we have already applied it. It should be some nice colour though” (FG3). Others explained, “I think if it has colour and it leaks, it won't look so pretty” (FG3); and, “It would be nice if they had various colours. I will not choose one without colour; I prefer a colour that looks strange and fun …” (FG2).

#### Taste: “A little sweet … is good”

Many participants preferred no taste: “I don't want taste; just scent is enough” (FG2). Some preferences reflected condom use for anal but not oral sex: “In my opinion, I don't want it with taste because when people have sex and use condoms, they do not use their mouth” (FG5). However, TG participants indicated: “Normally, lubricants have a bit of sweet taste at the tip of your tongue … so I think they should maintain a mild sweet taste like that” (FG3); “When we suck on a condom, it's a little sweet, which is good” (FG3).

### Individual

#### Sexual risk behaviour: “No condom, no worries”

Participants in every group described concerns that an RM “will create more HIV risk to people”: “from now on we have a gel available, and don't need condoms anymore” (FG2). Similarly, “Most gay people don't like to use condoms … if this gel is available it would be more convenient and they will use the gel” (FG4). Some comments about ceasing condom use reflected the contingency that an RM be completely effective: “If this rectal microbicide works 100%, then you meet a very handsome guy who happens to be positive; you can just use this on his dick and fuck. No condom, no worries, right?” (FG3); however others indicated, “If it's 50% effective I will choose to use the microbicide and not use a condom” (FG4). Some articulated a more cautious approach: “Smart people would still keep using condoms” (FG3).

#### Packaging/portability: “Maybe it can just look cool”

Preferences about RM packaging focused on detection and portability. Detection by others reflected concerns that an RM signifies sex or illness: “I want it disposable because if it was a big bottle, anybody else might know from its label … what the product was and think that we often had sex” (FG5); and, “What if your mother comes into your room and asks what it is?” (FG3). Participants articulated concerns about package appearance – “it should not look like a drug box; maybe it can just look cool …” (FG2) – and labelling – “I prefer a pack that says ‘microbicide lubricant’ but not ‘rectal microbicide’; if it came with the word rectal, people would think a lot before buying it” (FG5).

Portability emerged in preference for an RM that is, “Easy to carry around and throw away after use” (FG2), particularly important for those engaged in sex work: “We need to take it with us all the time” (FG5).

#### Timing/duration of protection: “Nobody will insert it before having sex”

Timing of RM use and duration of protection reflected challenges of pericoital use, multiple sexual encounters and cost, and were contingent on formulation. Challenges of pericoital use included interrupting or delaying sex: “if it is in suppository form, nobody will insert it before having sex” (FG4); “I am worried … if we use it by inserting, will it take a long time before it melts?” (FG2). Concerns that “using it before sex means having to start sex immediately after applying it” (FG4) resulted in preference for a once-daily product: “It would be better if it works like a birth control pill; if a woman takes one pill it can control for one day, right?” (FG2). Once-daily usage was preferred by those engaged in sex work: “It would be nice if it is one-day long because we don't have sex only one time … we have it two or three times per day” (FG4). Some participants preferred pericoital use, particularly for a gel: “It should be in the form of a gel used before having sex” (FG5).

### Interpersonal

#### Trust and communication: “It takes two to work it out”

Trust and communication includes challenges due to disclosure of RM use and signification of HIV risk and sex outside a primary relationship. Participants explained the importance of communication with primary partners about RM use: “… if we are lovers, we should talk” (FG1); “We have to come up with an agreement first that we will be using this RM” (FG3). One concern was that an RM might raise suspicion about HIV infection: “For sure sometimes the partner would say, ‘Why are you taking this with you? You have HIV disease?’” (FG2). A TG participant indicated risks with “straight guys”: “Talking about straight guys, not gay guys, they will not be able to accept this. They won't know; they may think that you have AIDS” (FG3). RMs also invoked mistrust: “if we have this, we will worry more about having sex with each other; will he use it with others or fuck around with others?” (FG2); “They always talk about trust and don't use condoms; if it was gel, there must be a reason why we would use it” (FG5).

#### Power/negotiation: “Don't ask … don't tell”

Power/negotiation includes rationales and strategies for enforcing RM use: assertion of the responsibility and right to protect oneself, to refuse unprotected sex and extolling the virtues of HIV prevention: “It is my personal thing; why do I have to care? I want to protect myself” (FG2); “No influence … it's our own safety!” (FG5). A TG sex worker asserted, “No acceptance, no fuck! Guys are everywhere” (FG3). Participants also discussed nondisclosure: “If they (sexual partners) don't ask, we don't tell; because we absolutely must use it” (FG1).

### Social–structural

#### Cost: “They should have both free and for sale”

Cost concerns entailed inability to afford an RM, appropriate pricing, reduced income from sex work and benefits of free products. Participants gauged appropriate cost in reference to condoms: “the price should be the same as a condom” (FG1); “… there are 3 condoms in one box, which costs 50–60 baht; compared to the microbicide, if it was too pricey, I wouldn't use it” (FG5). Similarly, “If it's a package for one time I think 20 baht is reasonable; it's not too expensive for something good for ourselves” (FG4). Sex workers lamented: “We gain a few hundred a day and then we have to spend more for having sex?” (FG3); and “They have to think of low-income TGs too … a onetime-use pack should cost like 5 THB” (FG3).

Participants described the need for free RMs at CBOs and public health venues to ensure access: “Like SWING, they hand out gel and condoms free” (FG4); “it should be free, but it would be better if located in Public Health” (FG2). Participants also noted, “they should have both free and for sale … sometimes the place that prorates is not open at night, so they should sell it at the 7–11 just like condoms” (FG2); though, “It shouldn't be too expensive because even condoms sold in markets are still expensive for people” (FG5).

#### Access: “We should be able to get it everywhere”

Participants generally indicated that requiring a prescription, hospital visit or to a lesser extent a pharmacy purchase would present barriers to accessing an RM. “I will give up if it requires a prescription from a doctor” (FG2); “it's too complicated to go to a hospital where doctors are always busy with many patients” (FG5). Rather, “we should be able to get it everywhere” (FG3). The array of venues included “Sell at 7-11” (FG1); “convenience stores like 7-11 that we can buy 24/7” (FG5); “vending machines in disco bathrooms” (FG2); “community organizations, clinics, hospitals and drug stores” (FG2).

#### Stigma: “It's like a sign on our forehead”

Stigma was associated with RMs as signifying HIV infection and being “dirty”: “The perception of people towards us is the thing; they would just think we are positive” (FG3); “they would think that we have HIV already; it's like a sign on our forehead” (FG5). Prescription requirements and hospital access would exacerbate stigma: “If we have to get it at hospitals, it would be such a horrible thing to get in there” (FG1); “For TGs to go to hospitals … not easy (giggle); just walking into one, we are stigmatized right away!” (FG3).

## Discussion

This investigation among gay and bisexual men and TG in Thailand reveals multi-level influences on future topical RM acceptability. In addition to product preferences for particular RM formulations and attributes, an array of individual, interpersonal and social–structural factors influenced product preferences and overall RM acceptability.

**Figure 1:**
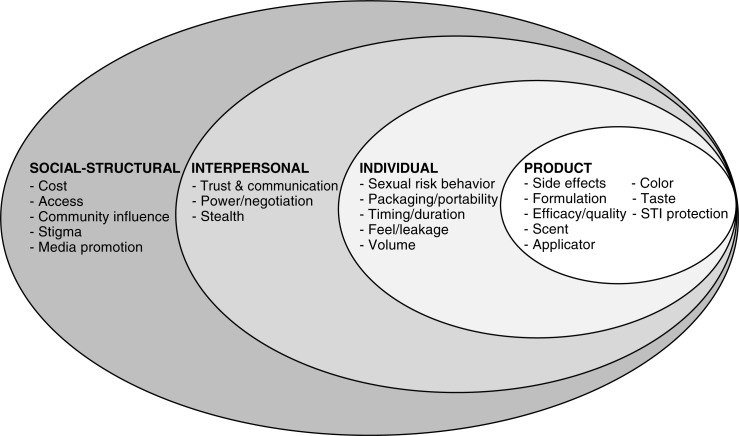
A social ecological model of rectal microbicide acceptability among gay/bisexual men and transgender women in Chiang Mai and Pattaya, Thailand (*n*=37). **STI, sexually transmitted infections**.

Figure 1 is a conceptual model that illustrates multi-level factors that impact upon RM acceptability. We embedded the product level, traditionally not a part of SEM, in the model guided by our focus on how participants’ social ecology may influence product acceptability and based on the data, which indicated multiple intersections between social ecological factors and product preferences.

The social–structural domain has been largely unexplored in regard to RMs, perhaps reflecting the relatively recent scientific focus on RM development. Yet, some advocates have assailed the decade-long delay in initiating research on microbicides designed for rectal use as a function of stigma and discrimination against gay men [33]. These same factors may engender challenges for future RM rollout. These findings suggest that intersecting influences of stigma and institutionalized discrimination against gay men/MSM and TG, venues for RM access, prescription requirements and cost-per-use may present obstacles to the effectiveness of future RMs outside of clinical trials, particularly among low-income MSM in LMICs. Interventions to mitigate stigma and discrimination against gay and other MSM and TG in the Thai health care system and broader society, and subsidies to support free and low-cost RMs in diverse venues may be fundamental to the successful contribution of RMs to controlling HIV on a population level.

Interpersonal concerns involved the impact of RM use on intimate relationships, differential challenges with primary, casual and paying partners, and the role of communication and power dynamics on RM implementation. Content analysis suggests greater interpersonal concerns among TG versus MSM, which may reflect intersecting factors on individual (TG are largely receptive partners), interpersonal (for some TG sexual acceptance from a male partner affirms their gender identification) and social–structural levels (some TG engage in sex work to pay for costly feminizing procedures) [25–27].

Individual-level concerns about the potential for RMs to exacerbate sexual risk indicate a role for educational interventions to be delivered in tandem with RMs, including information about partial efficacy and the need to sustain condom use. From a social ecological perspective, ostensibly individual-level concerns also evince a gap between what may be seen as a multiple-choice menu of “combination prevention” and the multi-layered, real-world challenges of implementing a complex array of partially efficacious and continually shifting prevention technologies [34]. The challenges of combination prevention may be compounded by structural inequities, such as poverty and low education; the escalating costs of safer sex may reduce feasibility among low-income MSM and TGs. Other individual-level themes – timing, duration of protection and portability – have similarly been identified among US MSM [8, 14–17]. The prevalence of packaging concerns, which we did not initially include in the interview guide, illustrates the value of a qualitative approach and SEM in contextualizing product preferences. Packaging concerns (i.e. product-level) reflect the importance of avoiding detection and stigma from parents, peers and sexual partners (i.e. interpersonal, social), which may be particularly relevant among young MSM and TGs, and in the Thai context (i.e. cultural). The Thai Buddhist tenet of *krengjai –* a pre-eminent value on maintaining interpersonal harmony and avoiding confrontation [24, 35] – may engender different socio-cultural challenges and opportunities than in the United States or elsewhere, and also may support culturally specific strategies to facilitate RM implementation in Thailand.

Product concerns largely corroborate findings from RM acceptability research among US MSM [8, 14–17]. The higher prevalence of product-level themes among TG versus MSM may reflect heightened concerns about side effects among receptive partners in anal sex and anticipated complications with feminizing hormones and medical procedures. The pervasive fear of side effects, particularly cancer, suggests the importance of advance product information and tailored education accessible to MSM and TGs with a 6th-grade education, and promotion from trusted Thai government and community public health sources. Herein product-level preferences intersect with individual (gender) and social–structural spheres of influence.

As a small qualitative study conducted among a convenience sample of young gay- and bisexual-identified MSM and TGs recruited from CBOs, the results may not be generalizable to other MSM and TGs in Thailand. MSM and TGs not connected with CBOs may harbour greater fears and misconceptions of HIV and RMs; and non-gay-identified MSM may face distinct RM challenges [22]. However, the shared findings from community participants in two cities recruited outside the selective realm of clinical trials broaden the applicability of the results. The group level of the data also limits our ability to assess individual differences in RM acceptability; by design, we assessed group differences by age and gender, but other demographic factors (e.g. education, income) also may influence acceptability. The small sample of TGs in one focus group also limits our ability to analyse possible within-population differences in RM acceptability. Nevertheless, as the first study of RM acceptability in Thailand, and the first we are aware of among MSM and TGs in an LMIC, we identified a social ecology of RM acceptability with multiple and intersecting levels of influence.

## Conclusions

Pervasive science-to-practice gaps across HIV preventive interventions – including male and female condoms, HIV testing and voluntary medical male circumcision [34] – and low adherence to microbicide use in clinical trials [5, 36], support the importance of conducting rigorous social science research in advance of RM availability that is designed to promote the transfer of biomedical and clinical RM research into routine health care practice and policy [9–12]. This study suggests that advancing an integrated implementation science approach in diverse socio-cultural contexts that acknowledges the intersecting influences of social–structural, interpersonal, individual-level and product-related factors may support the effectiveness of RMs in helping to control the HIV epidemic.
